# Multiomics Integrated Analysis Identifies *SLC24A2* as a Potential Link between Type 2 Diabetes and Cancer

**DOI:** 10.1155/2022/4629419

**Published:** 2022-05-13

**Authors:** Qin Bian, Haijun Li, Xiaoyi Wang, Tingting Liang, Kai Zhang

**Affiliations:** ^1^Department of Clinical Laboratory, Guangyuan Central Hospital, Guangyuan 628000, China; ^2^Department of Medical Imaging, Guangyuan Central Hospital, Guangyuan 628000, China; ^3^Department of Hospital-Acquired Infection Control, Guangyuan Central Hospital, Guangyuan 628000, China; ^4^School of Public Health, Shanghai Jiao Tong University School of Medicine, Shanghai 200000, China

## Abstract

**Background:**

So far, type 2 diabetes (T2D) is considered as an independent risk factor for various cancers, but the underlying mechanism remains unclear. *Methods. SLC24A2* was first identified as a key gene strongly associated with fasting plasma glucose (FPG). Then, overlapped differentially expressed genes (DEGs) between T2D verse control and *SLC24A2*-high verse *SLC24A2*-low were extracted and imported into weighted correlation network analysis. Gene Ontology, Kyoto Encyclopedia of Genes and Genomes, and gene set enrichment analysis were used for functional enrichment analysis of DEGs. Least absolute shrinkage and selection operator was utilized to build a T2D prediction model. Timer and *K*-*M* plotters were employed to find the expression and prognosis of *SLC24A2* in pan cancer.

**Results:**

Interestingly, both DEGs between T2D verse control and *SLC24A2*-high verse *SLC24A2*-low enriched in cancer-related pathways. Moreover, a total of 3719 overlapped DEGs were divided into 8 functional modules. Grey module negatively correlated with T2D and FPG and was markedly involved in ribosome biogenesis. Ten *SLC24A2*-related genes (*RRP36*, *RPF1*, *GRWD1*, *FBL*, *EXOSC5*, *BCCIP*, *UTP14A*, *TWISTNB*, *TBL3*, and *SKIV2L*) were identified as hub genes, based on which the LASSO model accurately predicts the occurrence of T2D (AUC = 0.841). In addition, *SLC24A2* was only expressed in islet *β* cells and showed abnormal expression in 17 kinds of cancers and significantly correlated with the prognosis of 10 kinds of cancers.

**Conclusion:**

Taken together, *SLC24A2* may link T2D and cancer by influencing the ribosome function of islet *β* cells and play different prognostic roles in different cancers.

## 1. Introduction

Type 2 diabetes mellitus (T2D) is a common chronic disease characterized by a high incidence rate, high disability rate, and high mortality worldwide. The World Health Organization (WHO) reported that diabetes will become the seventh leading cause of death by 2030. Epidemiological studies suggest that T2D was associated with various cancers such as breast cancer [1], liver cancer [2], lung cancer, pancreatic cancer, and prostate cancer [3–5]. For one thing, the incidence of cancer in T2D patients was higher than that in nondiabetic patients [6, 7]. Diabetes was also an independent poor prognostic factor of several cancers [8, 9]. For another, cancers and anticancer drugs lead to several adverse effects in T2D patients, such as microalbuminuria, diabetic retinopathy, and other acute diseases [10, 11]. Besides, a key gene, insulin receptor, mainly regulates the transformation of diabetes to cancer by enhancing insulin sensitivity, mediating antiapoptotic effects through combining with insulin growth factor 2 (*IGF2*), and leading to drug resistance in immunotherapy in immunotherapy [12–14]. Hence, it is urgent to find biomarkers and targets for early diagnosis of T2D.

Solute carrier (SLC) family, the second largest membrane protein family in human, is mainly responsible for the absorption and transportation of amino acids, nucleotides, glucose, inorganic ions, and drugs in the cell membrane [15–17]. *SLC24A2* is a new Na/Ca exchanger, which has the ability to regulate calcium homeostasis in mammalian cells or tissues [18]. Studies have shown that calcium channels on the islet *β* cell membrane regulated intracellular calcium signals, thereby affecting insulin secretion, which were closely related to the occurrence, development, and treatment of diabetes [19]. However, the role of *SLC24A2* in diabetes remains unclear.

To further clarify the function of *SLC24A2* in T2D and cancer, we comprehensively analyzed the expression and function of *SLC24A2* and its related genes in multiple datasets through bioinformatics methods, constructed an efficient T2D prediction model using lasso algorithm, and then evaluated the correlation with cancer prognosis, which reveal the potential mechanism linking T2D and cancers and provided a new target for the diagnosis and treatment of T2D.

## 2. Materials and Methods

### 2.1. Data Collection and Preprocessing

The transcriptome and clinical data of 4 type 2 diabetes- (T2D-) related datasets (GSE76896, GSE20966, GSE25724, and GSE154126) were downloaded from the Gene Expression Omnibus (GEO) database. GSE76896 based on the GPL570 platform consists of pancreatic samples from 55 diabetic (T2D) patients and 116 nondiabetic (ND) patients. Pancreases of 10 control and 10 T2D subjects from GSE20966 based on the GPL1352 platform were obtained. GSE25724 based on the GPL96 platform consists of human islets of 6 T2D patients and 7 ND subjects. Affymetrix Human Genome U133 Plus 2.0 Array (HG-U133_Plus_2), Affymetrix Human X3P Array (U133_X3P), and Affymetrix Human Genome U133A (HG-U133A) Array platform annotation information was used to annotate genes. Single-cell RNA-seq data of islets of T2D donors were obtained from GSE154126. The “limma” [20] *R* package was utilized to identify the differentially expressed genes (DEGs) between both T2D verse control and *SLC24A2*-high T2D verse *SLC24A2*-low T2D patients with a significance threshold of adjusted *P* < 0.05 and |logFC| > 1.0.

### 2.2. Weighted Gene Coexpression Network Analysis (WGCNA)

The overlapped DEGs in T2D verse control and *SLC24A2*-high T2D verse *SLC24A2*-low T2D patients were utilized to construct the weighted gene coexpression network analysis (WGCNA) with the “WGCNA” [21] *R* package. Firstly, the hierarchical clustering analysis of all genes was performed by hclust function. Then, the soft threshold was filtered with picki soft threshold function and selected when the independence was greater than 0.8. The correlation between gene module and clinical information was calculated. The minimum number of genes in the module was set to 30. A unique color label was assigned to each module.

### 2.3. Identification of Hub Genes

The gene contained in the grey module, which is the most closely related to T2D, was introduced into the STRING (http://www.string-db.org) database to construct the protein-protein interaction network. After that, the hub gene of the grey module was identified with “Cytohubba” [22] application of Cytoscape software (version 3.8.0).

### 2.4. Gene Set Enrichment Analysis

Gene Ontology (GO), Kyoto Encyclopedia of Genes and Genomes (KEGG), and Gene Set Enrichment Analysis (GSEA) were analyzed using “clusterProfiler” [23] *R* package. The c5.bp.v7.3.symbols.gmt and c2.cp.kegg.v7.3.symbols.gmt in the MsigDB V7.2 database (http://www.gsea-msigdb.org/gsea/msigdb/) were used as reference genesets.

### 2.5. Construction of LASSO Model and Receiver Operating Characteristic (ROC) Curve Analysis

Least absolute shrinkage and selection operator (LASSO) was constructed by “glmnet” [24] *R* package. According to the obtained regression coefficients, a model index was created for each sample. The weighted expression value of all selected genes was calculated as the following formula:index = ^“^ExpGene1 × Coef1 + ExpGene2 × Coef2 + ExpGene3 × Coef3 + .⋯^”^. The “Coef” derived from lasso Cox regression represents the regression coefficient of each gene, while “Exp” means the mRNA expression value of each gene. Then, GSE76896 dataset was randomly divided into training set (70%) and test set (30%). To evaluate the performance of LASSO model to recognize T2D independently, the ROC curves of the training set, test set, and *SLC24A2* alone were analyzed by using “pROC” [25] *R* package.

### 2.6. Bioinformatics Analysis

The expression and prognosis of *SLC24A2* in pan-cancer were analyzed by the TIMER 2.0 database (http://timer.comp-genomics.org/) and Kaplan-Meier plotter (http://www.kmplot.com/analysis/index.php?p=background), respectively.

### 2.7. Statistical Analysis

The “ggstatsplot” [26] *R* package was utilized to analyze the correlation between *SLC24A2* and FPG in T2D patients. The “ggcorrplot” [27] *R* package was used to analyze the correlation among 10 hub genes in the grey module. The comparison between *SLC24A2* in T2D and non-T2D was analyzed by two independent sample *t*-tests. *P* < 0.05 was considered statistically significant.

## 3. Results

### 3.1. Identification of *SLC24A2* or T2D-Related Differentially Expressed Genes (DEGs)

**Figure 1:**
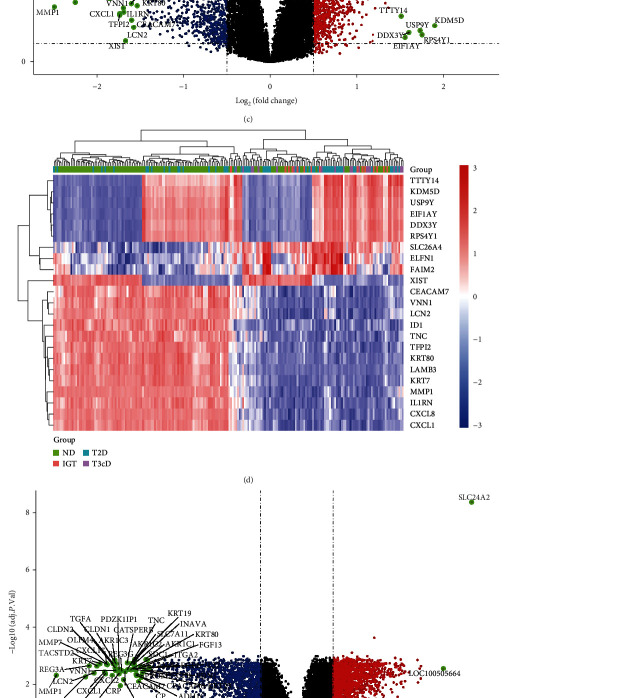
Identification of differentially expressed genes. (a) *SLC24A2* is upregulated in T2D (logFC = 1.023, *P* = 3.01*e* − 03). (b) *SLC24A2* is negatively correlated with fasting plasma glucose. (c) Volcano plot of the T2D verse control, red is the upregulated gene, blue is the downregulated gene, and black is not a significantly differentially expressed genes. (d) Heatmap of differentially expressed genes with |logFC| > 1.5 in T2D verse control. (e) Volcano plot of *SLC24A2*-high verse *SLC24A2*-low, red is the upregulated gene, blue is the downregulated gene, and black is not a significantly differentially expressed genes. (f) Heatmap of differentially expressed genes with |logFC| > 1.5 in *SLC24A2*-high verse *SLC24A2*-low.

In the GSE76896 dataset, the mRNA expression level of *SLC24A2* in the T2D group was significantly higher than that in the non-T2D group (logFC = 1.023, *P* = 3.01*e* − 03) (Figure 1(a)) and significantly negatively correlated with fasting plasma glucose (FPG) level (*r* = −0.43, *P* = 0.002) (Figure 1(b)). Compared with healthy individuals, 7108 differentially expressed genes (DEGs) were identified in the T2D group, of which 3576 were upregulated and 3532 were downregulated (Figure 1(c)). The heat map showed all the gene expression with |logFC| > 1.5 in T2D verse and non-T2D group (Figure 1(d)). In addition, compared with the *SLC24A2*-low group, a total of 5952 DEGs were identified in the *SLC24A2*-high group, including 3414 upregulated genes and 2538 downregulated genes (Figure 1(e)). The heat map showed all the gene expression with |logFC| > 1.5 in the *SLC24A2*-high verse *SLC24A2*-low group (Figure 1(f)). Finally, a total of 3719 overlapped genes were obtained in the T2D/non-T2D group and *SLC24A2*-high/*SLC24A2*-low group, which were considered as both *SLC24A2-* and T2D-related genes.

### 3.2. Weighted Correlation Network Analysis (WGCNA) Identifies T2D-Related Modules

**Figure 2:**
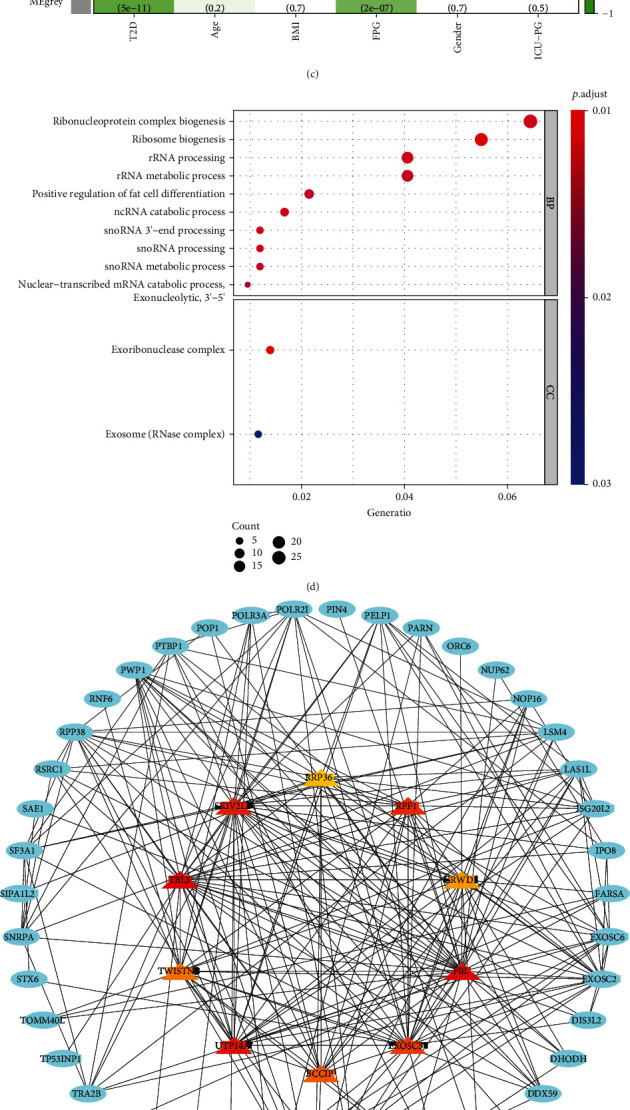
Weighted correlation network analysis. (a) Soft threshold selection. (b) Identification of 8 gene modules with WGCNA. (c) Correlation heat map of modules and phenotypes, red represents positively correlated with the phenotype, and green represents negatively correlated with the phenotype. (d) GO analysis of genes included in the grey module. (e) Protein-protein interaction of genes included in the grey module. (f) Correlation between *SLC24A2* and its related 10 hub genes, green indicates negative correlation, and yellow indicates positive correlation. GO: Gene Ontology.

To identify the key modules related to T2D, WGCNA was performed to analyze the mRNA expression matrix of the 3719 DEGs obtained in the previous step. The results showed that a total of 8 modules (Figure 2(b)) were identified when 17 was selected as the optimal soft threshold (Figure 2(a)). Grey module was negatively correlated with disease type (*r* = −0.48, *P* = 5*e* − 11) and fasting blood glucose (FPG) (*r* = −0.38, *P* = 2*e* − 07), while turquoise module was positively correlated with disease type (*r* = 0.36, *P* = 2*e* − 06) and FPG (*r* = 0.31, *P* = 3*e* − 05) (Figure 2(c)). In addition, the green module was also negatively correlated with T2D (*r* = −0.41, *P* = 3*e* − 08) and FPG (*r* = −0.34, *P* = 4*e* − 06) (Figure 2(c)). Next, the genes contained in the grey module, which is the most relevant to T2D and FPG, were selected for further analysis. GO enrichment analysis results showed that genes in the grey module were significantly enriched in several biological processes, such as ribonucleoprotein complex biogenesis, ribosome biogenesis, and positive regulation of fat cell differentiation, and mainly participated in cellular components, such as exoribonuclease complex and exosome (Figure 2(d)). Finally, the Cytohubba algorithm was used to identify 10 hub genes, including *RRP36*, *RPF1*, *GRWD1*, *FBL*, *EXOSC5*, *BCCIP*, *UTP14A*, *TWISTNB*, *TBL3*, and *SKIV2L* (Figure 2(e)). The correlation analysis showed an extensive regulatory relationship between 10 hub genes and *SLC24A2* (Figure 2(f)).

### 3.3. Gene Set Enrichment Analysis (GSEA) of *SLC24A2* or T2D-Related DEGs

**Figure 3:**
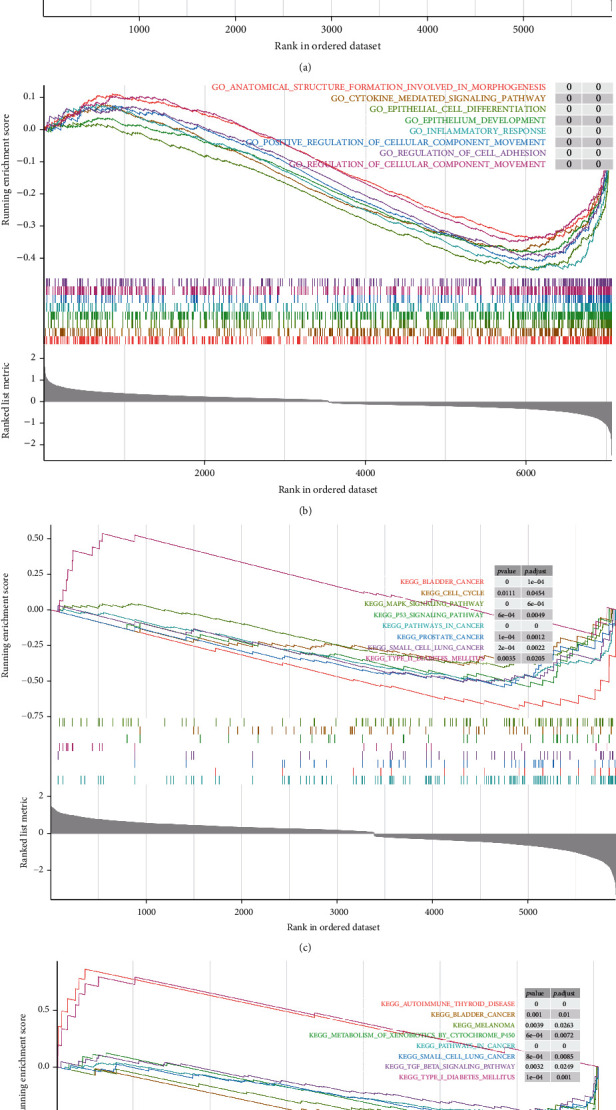
Gene set enrichment analysis. (a) GO-biological processes enriched in T2D. (b) GO-biological processes enriched in *SLC24A2*-high. (c) KEGG pathways enriched in T2D. (d) KEGG pathways enriched in *SLC24A2*-high. GO: Gene Ontology; KEGG: Kyoto Encyclopedia of Genes and Genomes.

Gene set enrichment analysis (GSEA) showed that compared with the *SLC24A2*-low group, the DEGs in the *SLC24A2*-high group were mainly enriched in some biological processes including anatomical structure formation involved in morphogenesis, animal organ morphogenesis, antimicrobial humoral response, and biological adhesion (Figure 3(a)) and several KEGG pathways, such as bladder cancer, pathways in cancer, prostate cancer, small-cell lung cancer, and type II diabetes mellitus (Figure 3(c)). Similarly, compared with the control group, the DEGs in the T2D group were significantly enriched in several biological processes, such as anatomical structure formation involved in morphogenesis, cytokine-mediated signaling pathway, epithelial cell differentiation, and epithelium development (Figure 3(b)) and notably enriched in pathways in cancer, small-cell lung cancer, bladder cancer, autoimmune thyroid disease, and type I diabetes mellitus KEGG pathways (Figure 3(d)). Interestingly, they shared anatomical structure formation involved in morphogenesis biological process and 4 cancer or diabetes-related KEGG pathways including pathways in cancer, bladder cancer, small-cell lung cancer, and type I/II diabetes mellitus, suggesting that *SLC24A2* may be a linkage between T2D and multiple cancers.

### 3.4. The Cell Source of *SLC24A2* and Hub Genes Was Determined with scRNA-seq Dataset

**Figure 4:**
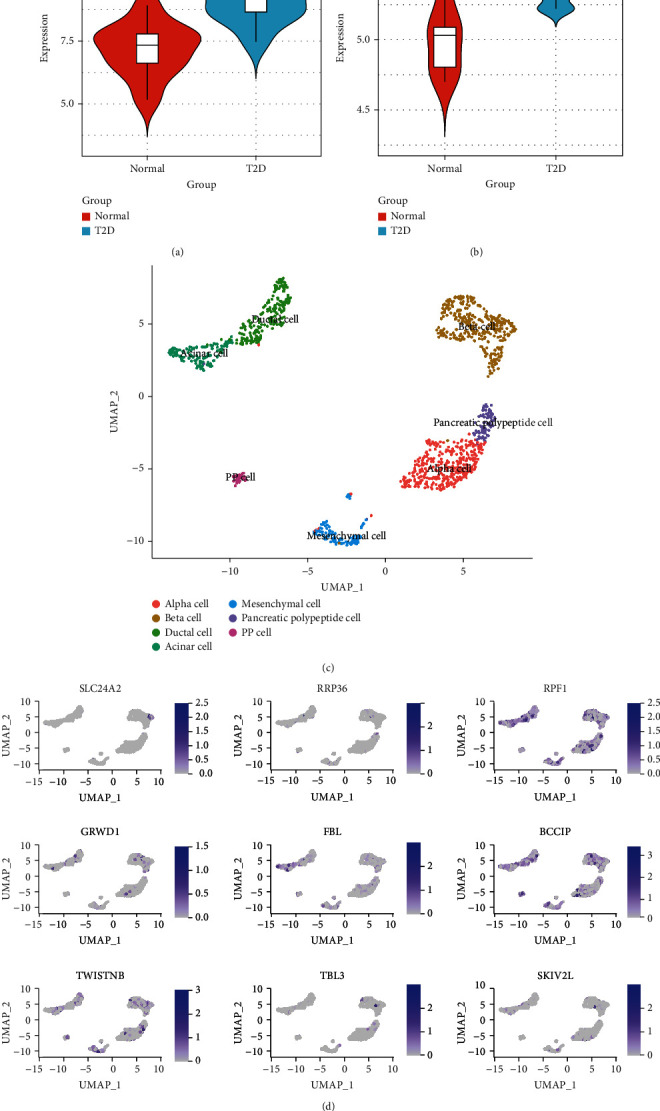
The *SLC24A2* expression validation on scRNA-seq dataset. (a) The *SLC24A2* expression between T2D and control in GSE20966. (b) The *SLC24A2* expression between T2D and control in GSE25724. (c) Cell cluster annotation with UMAP dimension reduction in GSE154126. (d) Feature plot of *SLC24A2* and 8 related hub gene expressions in GSE154126.

The higher expression of *SLC24A2* in T2D patients was significantly observed in GSE20966 (*P* = 0.0011) and GSE25724 (*P* = 0.0023) datasets (Figures 4(a) and 4(b) ). After excluding cells with the mitochondrial gene expression ratio more than 5%, all cells from T2D samples were clustered into 7 clusters (islet *α* cells, islet *β* cells, ductal cells, acinar cells, mesenchymal cells, pancreatic polypeptide cells, and PP cells) by UMAP dimension reduction method in the GSE154126 dataset (Figure 4(c)). Results showed that *SLC24A2* and its related 8 hub genes were all expressed in islet *β* cells (Figure 4(d)).

### 3.5. The LASSO Model Is an Effective Prediction Tool for T2D

**Figure 5:**
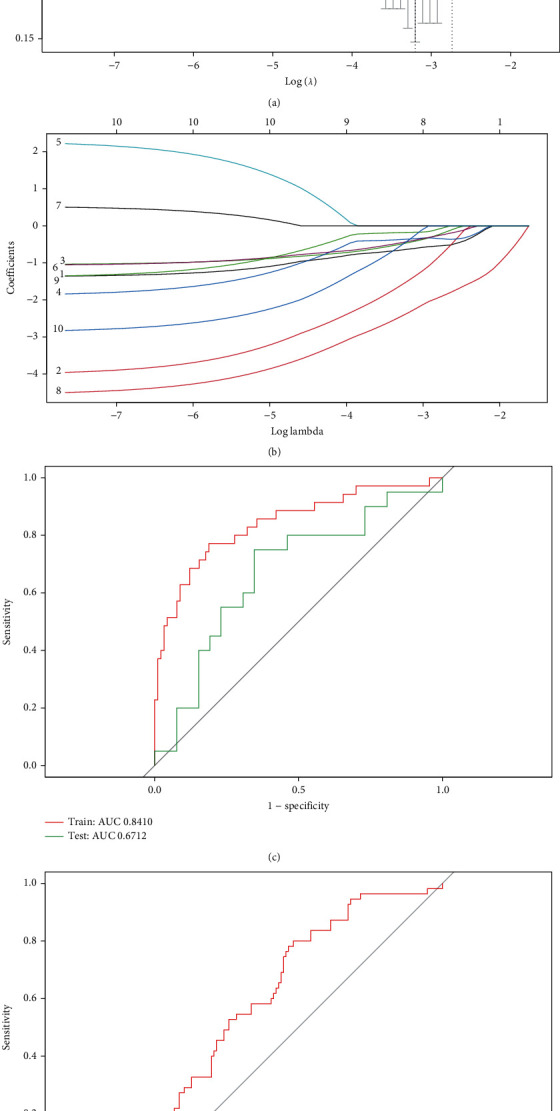
The LASSO model for predicting T2D. (a, b) LASSO model. (c) ROC analysis of train set and validation set. (d) ROC analysis of *SLC24A2*. LASSO: least absolute shrinkage and selection operator; ROC: receiver operating characteristic curve.

Firstly, the expression matrices of 10 hub genes were extracted. After calculation by the LASSO algorithm, the nonzero regression coefficients of 8 genes were obtained, and the value of lambda.min is 0.04065766 (Figures 5(a) and 5(b)). The detailed risk score is calculated as the following formula: index = RRP36 × (−0.6333954) + RPF1 × (−1.4930099) + GRWD1 × (−0.4587730) + *FBL* × (−0.3610982) + *BCCIP* × (−0.4258075) + *TWISTNB* × (−2.3570415) + TBL3 × (−0.1784551) + SKIV2L × (−0.4068790). ROC curve showed that the AUC of *SLC24A2* for independently predicting T2D was 0.6856 (Figure 5(d)), while 8-gene model reached 0.8410 in the training set and 0.6712 in the test set, indicating that the LASSO model may be an efficient tool to explore early biomarkers for diagnosis of T2D (Figure 5(c)).

### 3.6. Pan-Cancer Analysis of *SLC24A2*

**Figure 6:**
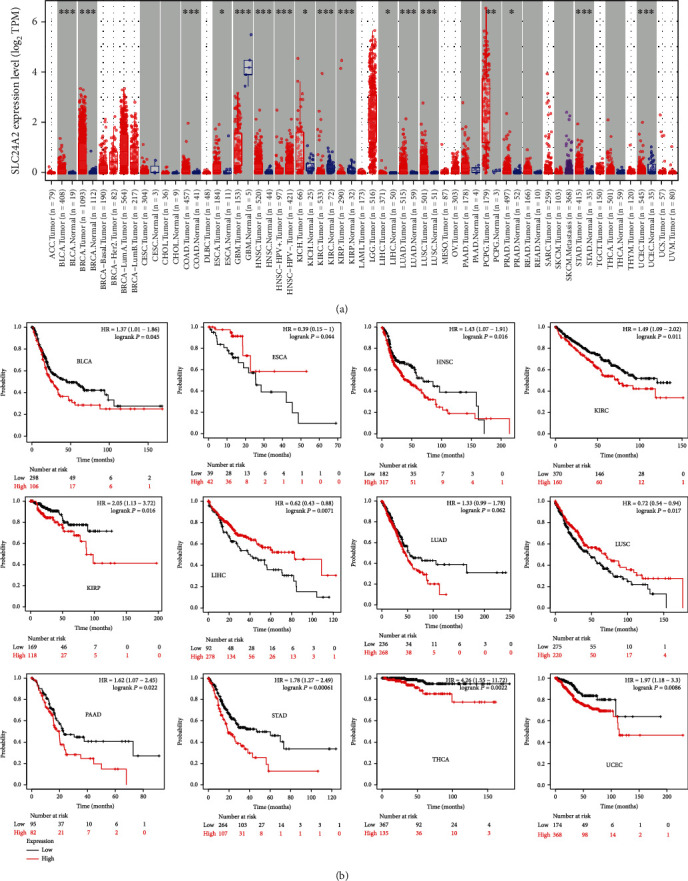
Expression and prognostic analysis of *SLC24A2* in pan cancer. (a) The expression of *SLC24A2* in pan cancer. (b) Prognostic analysis of *SLC24A2* in pan cancer.

Consistent with previous enrichment analysis of DEGs, *SLC24A2* showed the abnormal expression in 17 cancers including bladder cancer (BLCA), prostate cancer (PRAD), and lung cancer (LUAD and LUSC) (Figure 6(a)). In detail, *SLC24A2* was notably lower expressed in glioblastoma (GBM), kidney renal clear cell carcinoma (KIRC), and kidney renal papillary cell carcinoma (KIRP) (all *P* < 0.05) than in adjacent tissues (Figure 6(a)), whose lower expression significantly correlated with high survival time in KIRC (HR = 1.49, *P* = 0.011) and KIRP (HR = 2.05, *P* = 0.016) (Figure 6(b)). Additionally, *SLC24A2* observably elevated in other cancers (Figure 6(a)), which was significantly correlated with the poor prognosis of BLCA (HR = 1.37, *P* = 0.045), head and neck squamous cell carcinoma (HNSC) (HR = 1.43, *P* = 0.016), lung adenocarcinoma (LUAD) (HR = 1.33, *P* = 0.062), stomach adenocarcinoma (STAD) (HR = 1.78, *P* = 0.00061), and uterine corpus endometrial carcinoma (UCEC) (HR = 1.97, *P* = 0.00086) but significantly correlated with the good prognosis of patients with esophageal cancer (ESCA) (HR = 0.39, *P* = 0.044), liver hepatocellular carcinoma (LIHC) (HR = 0.62, *P* = 0.0071), and lung squamous cell carcinoma (LUSC) (HR = 0.72, *P* = 0.017) (Figure 6(b)). That is to say, although *SLC24A2* may play different roles in different cancers, they were all closely related to prognosis.

## 4. Discussion

As we all know that type 2 diabetes (T2D) is a common chronic disease, usually accompanied by a variety of complications including cancer, however, the association between T2D and cancer has not yet been fully elucidated. Meanwhile, this lacks effective biomarkers for early diagnosis of T2D. Increasing evidences have demonstrated that the SLC family plays a key role in the pathogenesis and diagnosis of both T2D and cancer [28–30]. However, the function of *SLC24A2* needs to be fully clarified and further explored.

Firstly, we found that *SLC24A2* was significantly upregulated in T2D patients within 3 datasets (GSE76896, GSE76896, and GSE76896) and negatively correlated with fasting plasma glucose (FPG) in GSE76896, suggesting that *SLC24A2* might be a potential inhibitor of T2D through lowering blood glucose. *SLC24A2*, also known as *NCKX2*, is a sodium/potassium/calcium exchanger of the solute carrier family, which was first reported to be associated with retinal diseases [18]. In recent years, *SLC24A2* has been found as a tumor microenvironment-related gene to be related to the prognosis of esophageal squamous cell carcinoma [31]. Nonetheless, no reports have confirmed the association between *SLC24A2* and T2D. It is well known that islet alpha cells and islet beta cells jointly regulate the level of blood glucose. In this study, scRNA-seq analysis revealed that *SLC24A2* was only expressed in islet beta cells, suggesting that *SLC24A2* may regulate blood glucose by affecting the function of islet beta cells. Therefore, this is the first study to clarify the potential mechanism of *SLC24A2* in the occurrence and development of T2D.

To further reveal the role of *SLC24A2* in the pathogenesis of T2D, we used the WGCNA algorithm to find key modules among DEGs both related to *SLC24A2* and T2D. WGCNA is an effective way to search for coexpressed gene modules and to explore the association between gene networks and different phenotypes, which has been applied to the exploration of key genes in various diseases including T2D [32–34]. On this basis, we further identified 10 hub genes by the Cytohubba algorithm, namely, *RRP36*, *RPF1*, *GRWD1*, *FBL*, *EXOSC5*, *BCCIP*, *UTP14A*, *TWISTNB*, *TBL3*, and *SKIV2L*. One study showed that the methylation level of *GRWD1* was associated with insulin resistance [35]. GWAS analysis revealed that *SKIV2L* was associated with inflammation status in patients with metabolic diseases such as diabetes [36]. Previous studies showed that *RRP36*, *RPF1*, *FBL*, and *BCCIP* affected ribosomal function and rRNA processing [37–39]. In addition, *EXOSC5*, *UTP14A*, *TWISTNB*, and *TBL3* were closely related to the occurrence or prognosis of several cancers [40–43]. Together with the results of GO enrichment analysis, we believed that abnormal ribosome function may be one of the causes of T2D complicated with cancer. Coincidently, Peng et al. also found that the shared DEGs of T2D and colorectal cancer (CRC) patients were significantly enriched in the ribosomal pathway in the study of T2D complicated with CRC patients [44].

Interestingly, we found broad correlations between 10 hub genes and *SLC24A2*, indicating that a comprehensive analysis of *SLC24A2*-related regulatory network members could serve as potential markers for early diagnosis of T2D. Therefore, the LASSO algorithm was introduced to further analysis. The LASSO algorithm could construct a penalty function to obtain a more refined model based on regularization, which has been widely used in the exploration of tumor biomarkers [45]. After that, an efficient 8-gene (*RRP36*, *RPF1*, *GRWD1*, *FBL*, *BCCIP*, *TWISTNB*, *TBL3*, and *SKIV2L*) T2D prediction model was obtained, whose AUC reached 0.84 and 0.67, respectively, in training set and test set. Thus, LASSO is a promising tool for early diagnosis of T2D and has a high potential value.

It is also noteworthy that the DEGs between high- and low- *SLC24A2* groups were enriched in T2D and several cancer pathways such as bladder cancer and non-small-cell lung cancer. Meanwhile, the DEGs between T2D and normal controls were also enriched in some cancer pathways including bladder cancer and non-small-cell lung cancer. That is to say, the shared DEGs imported into WGCNA, as well as *SLC24A2*, could explain the coexistence of T2D and cancer to some extent. Therefore, in addition to the analysis of PPI network and LASSO-prediction model for shared DEGs mentioned above, we also focused on the prognostic value of *SLC24A2* in pan cancers. The results showed that *SLC24A2* played opposite roles in different types of cancers due to the tissue specificity of *SLC24A2*.

In order to achieve better clinical application, we could make full use of *SLC24A2* and its related genes for early clinical diagnosis. In addition, we could develop *SLC24A2* inhibitors for clinical research. Although we explored the role of SC24A2 between T2D and cancers through a multiomics approach, this study still has some limitations. On the one hand, it is necessary to verify the prognostic value and robustness of the model in expanded samples. On the other hand, we also need to further verify the potential mechanism through in vivo and in vitro experiments to promote clinical application and transformation.

## 5. Conclusion

In summary, we used bioinformatics methods and multiple algorithms to integrate multiple RNA-seq datasets and scRNA-seq dataset and found that *SLC24A2* may prevent the occurrence of T2D complicated by cancer via maintaining the ribosome function of islet beta cells and play different prognostic roles in cancers.

## Data Availability

All data used in this study are from public databases, including GEO, TCGA, and *K*-*M* plotter. Detailed information could be found in the method section of the manuscript.
